# Aneurysm and Artery Dissection After Oral VEGFR-TKI Use in Adults With Cancer

**DOI:** 10.1001/jamanetworkopen.2023.45977

**Published:** 2023-11-29

**Authors:** Soyoung Kang, Bora Yeon, Myo-Song Kim, Myungsik Yoo, Bonggi Kim, Yun Mi Yu

**Affiliations:** 1 Department of Drug Safety Information, Korea Institute of Drug Safety and Risk Management, Gyeonggi-do, Republic of Korea; 2 Department of Pharmacy and Yonsei Institute of Pharmaceutical Sciences, College of Pharmacy, Yonsei University, Incheon, Republic of Korea; 3 Department of Pharmaceutical Medicine and Regulatory Sciences, College of Pharmacy, Yonsei University, Incheon, Republic of Korea

## Abstract

**Importance:**

The association of tyrosine kinase inhibitors targeting vascular endothelial growth factor receptors (VEGFR-TKIs) with aneurysm and artery dissection (AAD) has been frequently reported in spontaneous reporting databases.

**Objective:**

To investigate the risk and incidence of AAD occurrence in patients with cancer treated with oral VEGFR-TKIs, with capecitabine as an active comparator.

**Design, Setting, and Participants:**

This national, historical cohort study was conducted using national claims data from the National Health Insurance Service in Korea from 2007 to 2020, with a 1-year follow-up. Patients with cancer aged 40 years or older prescribed oral VEGFR-TKIs or capecitabine were enrolled. Data were analyzed from September 2022 through April 2023.

**Exposure:**

Oral VEGFR-TKIs (sorafenib, regorafenib, vandetanib, sunitinib, lenvatinib, axitinib, and pazopanib) or capecitabine as a comparator.

**Main Outcomes and Measures:**

Hazard ratios (HRs) were used to investigate the association between VEGFR-TKI use and AAD after propensity score matching. The primary outcome was AAD, and secondary outcomes were aortic aneurysm and dissection and AAD with rupture. Outcomes were defined using *International Statistical Classification of Diseases and Related Health Problems, Tenth Revision* (*ICD-10*) diagnosis codes.

**Results:**

Among 127 710 patients with cancer eligible for the study (80 386 males [62.9%]; mean [SD] age, 62.6 [10.9] years), 37 308 patients received VEGFR-TKIs and 90 402 patients received capecitabine. Among 27 535 matched patients receiving VEGFR-TKIs, the incidence of AAD within 1 year of treatment initiation was 6.0 per 1000 person-years. The median (IQR) time to AAD onset in the matched AAD group was 114 (67-257) days after treatment initiation, with the highest incidence observed during the first 3 months (45 incidents vs 31, 17, and 16 incidents during 3- to 6-month, 6- to 9-month, and 9- to 12-month periods, respectively). Cox regression modeling showed that the risk of AAD occurrence was significantly higher among patients prescribed VEGFR-TKIs than those receiving capecitabine (HR, 1.48; 95% CI, 1.08-2.02); similar results were obtained among females (HR, 2.08; 95% CI, 1.26-3.42), older adults (aged ≥65 years; HR, 1.42; 95% CI, 1.01-1.99), and patients with dyslipidemia (HR, 1.58; 95% CI, 1.11-2.24).

**Conclusions and Relevance:**

In this study, the use of oral VEGFR-TKIs was associated with an increased risk of AAD occurrence. These findings elucidate vascular toxic effects and may provide a substantial reference for reducing the socioeconomic burden of adverse events associated with VEGFR-TKI use.

## Key Points

**Question:**

Is treatment with oral tyrosine kinase inhibitors targeting vascular endothelial growth factor receptors (VEGFR-TKIs) in patients with cancer associated with aneurysm and artery dissection (AAD)?

**Findings:**

In this cohort study of 127 710 patients with cancer aged 40 years and older, an association was identified between the use of oral VEGFR-TKIs and increased risk of AAD compared with the use of capecitabine. The incidence rate of AAD within 1 year of treatment initiation was 6.0 per 1000 person-years.

**Meaning:**

This study found an association between oral VEGFR-TKIs and vascular toxic effects, which may help to mitigate the socioeconomic burden of adverse events associated with use of these drugs.

## Teaser

This cohort study investigates the association of tyrosine kinase inhibitors targeting vascular endothelial growth factor receptors with aneurysm and artery dissection in patients with cancer.

## Introduction

To minimize systemic toxic effects associated with traditional anticancer drugs,^1^ various targeted therapies have been developed to deliver drugs to specific genes or enzymes that promote cancer cell growth in the tumor microenvironment.^2,3^ Angiogenesis, the formation of new blood vessels, influences cancer growth and metastasis.^4^ The vascular endothelial growth factor (VEGF) is a key mediator of angiogenesis. Tyrosine kinase inhibitors targeting the VEGF receptor (VEGFR-TKIs) effectively suppress angiogenesis and are used in the treatment of metastatic cancers.^5,6^

VEGFR-TKIs are associated with adverse outcomes, such as hypothyroidism, delayed wound healing, gastrointestinal perforation, and proteinuria, which are rarely reported with traditional anticancer drugs.^7,8,9^ Additionally, the VEGF pathway is involved in angiogenesis, endothelial cell integrity, and vascular tone, and so adverse outcomes, such as hypertension, arterial thromboembolism, and left ventricular dysfunction, have also been reported after treatment with VEGFR-TKIs.^10,11,12,13^

An association between VEGF pathway inhibitors and the incidence of aneurysm and artery dissection (AAD) has been reported.^14,15,16,17,18^ Consequently, regulatory agencies, such as the US Food and Drug Administration and UK Medicines and Healthcare Products Regulatory Agency, promptly updated the labeling of VEGFR-TKIs to reflect this association and urged health care professionals to use caution in administration of these inhibitors to patients with risk factors, such as hypertension and a history of arterial aneurysm, smoking, and diabetes.^19,20^ However, most studies based on spontaneous reporting databases had some limitations, including underreporting, subjective and imprecise adverse event recognition, low report quality, and a lack of denominator data (ie, population exposure).^21^ To our knowledge, no epidemiological study to date on patients receiving VEGFR-TKIs has investigated incidence rates and risk factors associated with AAD based on clinical data. Therefore, this study evaluated the association between the use of VEGFR-TKIs and AAD occurrence, calculated incidence rates of AAD, and identified associated risk factors.

## Methods

### Study Design and Data Source

The protocol for this cohort study was approved by the institutional review board of the Korea Institute of Drug Safety (KIDS) and Risk Management and adhered to the ethical principles set by the Declaration of Helsinki. The study was performed according to Strengthening the Reporting of Observational Studies in Epidemiology (STROBE) reporting guideline. The requirement for written informed consent was waived by KIDS owing to the historical study design.

This historical cohort study used nationwide claims data of the National Health Insurance Service (NHIS) between January 1, 2007, and December 31, 2021, given that this period covers the largest number of patients using VEGFR-TKIs. The NHIS database contains claims data for approximately 98% of the national population of South Korea^22^ and includes demographic and clinical information, such as sex, dates of birth and death, diagnoses, procedures, and prescription data from submitted claims. All personal information was anonymized according to confidentiality regulations of the NHIS.

### Study Population and Drugs

To assess the risk of AAD occurrence in patients who had received treatment for 1 year, patients aged 40 years and older newly prescribed oral VEGFR-TKIs (ie, sorafenib, regorafenib, vandetanib, sunitinib, lenvatinib, axitinib, and pazopanib) as study drugs or capecitabine as a comparator drug between January 1, 2010, and December 31, 2020, were enrolled. To identify new users of VEGFR-TKIs after 2010, patients with prescription records of VEGFR-TKIs between January 1, 2007, and December 31, 2009, were excluded. Patient enrollments were limited to those during 2020 to ensure a 1-year follow-up. The date of the first prescription of VEGFR-TKIs or capecitabine during the available data period was considered the index date.

We excluded patients prescribed VEGF pathway inhibitors that were administered orally (ie, bevacizumab, ziv-aflibercept, and ramucirumab) or used for the treatment of solid tumors (ie, ponatinib, aflibercept, ranibizumab, and brolucizumab) from 3 years before the index date to the censoring date. Patients prescribed nintedanib were excluded owing to the lack of NHIS claims data, while those prescribed cabozantinib were excluded because this drug is used as secondary treatment after treatment with other oral VEGFR-TKIs based on drug approval labeling in South Korea. The exclusion of patients prescribed cabozantinib was expected to have minimal impact on our results because cabozantinib is an orphan drug with low usage. Patients diagnosed with AAD before or on the index date and those with congenital diseases related to arterial aneurysm were excluded (eTable 1 in Supplement 1).^23,24,25^ Diagnoses were based on *International Statistical Classification of Diseases and Related Health Problems, Tenth Revision* (*ICD-10*) codes. Lastly, patients prescribed both VEGFR-TKIs and capecitabine were excluded. The study design is illustrated in eFigure 1 in Supplement 1.^26^

### Outcomes

The primary outcome, AAD with or without rupture, and secondary outcomes, aortic aneurysm and dissection and AAD with rupture, were defined using *ICD-10* diagnosis codes during the 1-year follow-up (eTable 1 in Supplement 1). Follow-up was performed for all participants from the index date to the first diagnosis of an outcome, death, or 1 year after the cohort entry date, whichever occurred first.

### Covariates and Data Collection

Demographic information, including sex and age, was recorded on the index date. Medical history,^25,27,28,29^ medication history,^23,30^ and anticancer therapy administered within a year prior to enrollment were recorded (eTable 2 in Supplement 1). Data on cancer subtypes as diagnosed on the index date were collected.

### Statistical Analysis

We performed 1:1 propensity score (PS) matching between patients prescribed VEGFR-TKIs and those prescribed capecitabine using a greedy matching algorithm^31^; a caliper width of 0.2 of the SD of the logit PS was applied.^32^ The PS was calculated using a multivariable logistic regression model that integrated demographic characteristics, medical and medication history, and history of anticancer therapy (Table 1); no missing data were identified for variables obtained from eligible patients. The matching of variables was considered balanced when the standardized difference between groups was less than 10%.^33^ The distribution of PSs between patients administered VEGFR-TKIs and those administered capecitabine was graphically inspected.

**Table 1.** Baseline Characteristics of Study Participants

Characteristic	Before PS matching			After PS matching		
	Patients, No. (%) (N = 127 710)		Standardized difference, %^a^	Patients, No. (%) (N = ‭55 070)		Standardized difference, %^a^
	VEGFR-TKI (n = 37 308 [29.2%])	Capecitabine (n = 90 402 [70.8%])		VEGFR-TKI (n = 27 535 [50.0%])	Capecitabine (n = 27 535 [50.0%])	
Sex						
Male	29 408 (78.8)	50 978 (56.4)	49.4	20 170 (73.3)	19 818 (72.0)	2.9
Female	7900 (21.2)	39 424 (43.6)		7365 (26.7)	7717 (28.0)	
Age, y						
Mean (SD)	62.4 (10.5)	62.7 (11.0)	4.0	62.9 (10.7)	63.1 (10.7)	1.3
40-49	4389 (11.8)	11 817 (13.1)		3167 (11.5)	3057 (11.1)	
50-59	11 291 (30.3)	24 913 (27.6)		7824 (28.4)	7738 (28.1)	
60-69	11 457 (30.7)	26 536 (29.4)		8431 (30.6)	8373 (30.4)	
≥70	10 171 (27.3)	27 136 (30.0)		8113 (29.5)	8367 (30.4)	
Medical history						
Acute coronary syndrome	1312 (3.5)	3315 (3.7)	0.8	1045 (3.8)	1015 (3.7)	0.6
Other ischemic heart disease	5291 (14.2)	12 331 (13.6)	1.6	4068 (14.8)	4027 (14.6)	0.4
Heart failure or cardiomyopathy	3307 (8.9)	7125 (7.9)	3.6	2474 (9.0)	2450 (8.9)	0.3
Valve disorder	158 (0.4)	482 (0.5)	1.6	128 (0.5)	131 (0.5)	0.2
Cerebrovascular disease	3299 (8.8)	8038 (8.9)	0.2	2625 (9.5)	2624 (9.5)	<0.1
Arterial disease	4544 (12.2)	11 131 (12.3)	0.4	3585 (13.0)	3596 (13.1)	0.1
Arrhythmia	2268 (6.1)	5433 (6.0)	0.3	1736 (6.3)	1710 (6.2)	0.4
Lung disease	12 984 (34.8)	33 276 (36.8)	4.2	10 100 (36.7)	10 388 (37.7)	2.2
Liver disease	29 504 (79.1)	40 727 (45.1)	74.9	19 808 (71.9)	19 171 (69.6)	5.1
Kidney disease	3731 (10.0)	5415 (6.0)	14.8	2601 (9.4)	2676 (9.7)	0.9
Rheumatic disease	1494 (4.0)	3389 (3.7)	1.3	1183 (4.3)	1174 (4.3)	0.2
Psychiatric disorder	10 035 (26.9)	27 427 (30.3)	7.6	8196 (29.8)	8290 (30.1)	0.8
Hypertension	19 375 (51.9)	39 802 (44.0)	15.9	13 852 (50.3)	14 022 (50.9)	1.2
Diabetes	15 942 (42.7)	33 217 (36.7)	12.3	11 630 (42.2)	11 556 (42.0)	0.5
Dyslipidemia	19 577 (52.5)	45 909 (50.8)	3.4	14 935 (54.2)	14 809 (53.8)	0.9
Trauma	14 085 (37.8)	29 625 (32.8)	10.4	10 083 (36.6)	10 208 (37.1)	0.9
Drug abuse	31 (0.1)	53 (0.1)	0.9	21 (0.1)	18 (0.1)	0.4
Obesity	14 (0.04)	64 (0.07)	1.4	13 (0.05)	13 (0.05)	<0.1
Medication history						
ACE inhibitor or ARB	11 629 (31.2)	24 888 (27.5)	8.0	8619 (31.3)	8707 (31.6)	0.7
Calcium channel blocker	14 621 (39.2)	33 466 (37.0)	4.5	10 873 (39.5)	10 978 (39.9)	0.8
Loop diuretic	11 879 (31.8)	26 434 (29.2)	5.7	8572 (31.1)	8665 (31.5)	0.7
Other diuretic	10 040 (26.9)	15 016 (16.6)	25.2	6295 (22.9)	6350 (23.1)	0.5
β-Blocker	9206 (24.7)	20 225 (22.4)	5.4	6519 (23.7)	6430 (23.4)	0.8
Digoxin	300 (0.8)	914 (1.0)	2.2	254 (0.9)	253 (0.9)	<0.1
Nitrate	4195 (11.2)	7563 (8.4)	9.7	2826 (10.3)	2884 (10.5)	0.7
Platelet inhibitor	7198 (19.3)	17 420 (19.3)	0.1	5680 (20.6)	5702 (20.7)	0.2
Anticoagulant	11 381 (30.5)	33 800 (37.4)	14.6	8703 (31.6)	8311 (30.2)	3.1
Lipid-lowering drug	6943 (18.6)	21 806 (24.1)	13.5	5911 (21.5)	5974 (21.7)	0.6
Antidiabetic drug (not insulin)	9069 (24.3)	16 984 (18.8)	13.5	6360 (23.1)	6383 (23.2)	0.2
Insulin	6623 (17.8)	13 627 (15.1)	7.2	4768 (17.3)	4712 (17.1)	0.5
Antidepressant	4340 (11.6)	11 953 (13.2)	4.8	3556 (12.9)	3584 (13.0)	0.3
Antipsychotic	1877 (5.0)	4061 (4.5)	2.5	1440 (5.2)	1495 (5.4)	0.9
Anxiolytic, hypnotic, or sedative	20 535 (55.0)	69 551 (76.9)	47.5	17 849 (64.8)	17 233 (62.6)	4.7
Corticosteroid for systemic use	20 838 (55.9)	52 828 (58.4)	5.2	15 942 (57.9)	16 269 (59.1)	2.4
NSAID	29 853 (80.0)	74 310 (82.2)	5.6	22 351 (81.2)	22 451 (81.5)	0.9
Opiate	31 673 (84.9)	78 986 (87.4)	7.2	23 293 (84.6)	23 086 (83.8)	2.1
Systemic HT	273 (0.7)	1101 (1.2)	5.0	252 (0.9)	267 (1.0)	0.6
Fluoroquinolone	10 380 (27.8)	28 572 (31.6)	8.3	8120 (29.5)	8161 (29.6)	0.3
G-CSF	1233 (3.3)	6898 (7.6)	19.1	1196 (4.3)	1285 (4.7)	1.6
Fibrinolytic drug	128 (0.3)	230 (0.3)	1.6	90 (0.3)	91 (0.3)	0.1
History of anticancer therapy						
Cytotoxic agent	16 000 (42.9)	23 430 (25.9)	36.3	9293 (33.7)	9267 (33.7)	0.2
Targeted anticancer drug	1438 (3.9)	5115 (5.7)	8.5	1325 (4.8)	1462 (5.3)	2.3
Hormone treatment	194 (0.5)	5534 (6.1)	31.7	193 (0.7)	266 (1.0)	2.9
Cancer immunotherapy	14 (0.04)	15 (0.02)	1.3	11 (0.04)	9 (0.03)	0.4
Other	123 (0.3)	71 (0.1)	5.6	61 (0.2)	56 (0.2)	0.4
Radiation therapy	8207 (22.0)	11 379 (12.6)	25.1	4555 (16.5)	4651 (16.9)	0.9

Abbreviations: ACE, angiotensin-converting enzyme; ARB, angiotensin II receptor blocker; G-CSF, granulocyte colony-stimulating factor; HT, hormone therapy; NSAID, nonsteroidal anti-inflammatory drug; PS, propensity score; VEGFR-TKI, vascular endothelial growth factor receptor tyrosine kinase inhibitor.

^a^ Matching of variables was considered balanced when the standardized difference was less than 10%.

After PS matching was performed, Cox proportional hazard regression analyses were conducted to compare the risk of AAD occurrence and estimate the hazard ratio (HR) at 95% CI. To assess the validity of the proportional hazard assumption, the statistical association between Schoenfeld residuals and survival time was determined by plotting a log-minus-log survival curve.^34^ We compared cumulative incidence curves of AAD for patients administered VEGFR-TKIs or capecitabine using Kaplan-Meier survival analysis and the unadjusted log-rank test. Subgroup analyses were performed based on several factors, including sex, age (40-64 years or ≥65 years), presence or absence of underlying diseases (such as hypertension, diabetes, and dyslipidemia), and cancer subtype. Age was categorized based on 65 years, the age at which the risk of developing AAD significantly increases.^35,36^

Sensitivity analyses were conducted by changing the follow-up from 1 year to 3, 6, or 9 months; considering the medication discontinuation date as the end of follow-up (as-treated analysis); restricting the analysis to patients prescribed VEGFR-TKIs or capecitabine 2 or more times during follow-up; adjusting for hypertension, which is a risk factor associated with AAD and common adverse outcome associated with VEGFR-TKI administration^10,11,37^; and calculating the *E* value. To assess treatment-outcome association, the *E* value was defined as the minimum strength of association that an unmeasured confounder must have with treatment and outcome.^38^ It served as a metric for assessing the robustness of observed associations, providing insight into the potential impact of unmeasured confounding factors on study results.

Discrete data were compared using the χ^2^ test or Fisher exact test based on the expected frequency. Statistical analyses were performed using SAS statistical software version 9.4 (SAS Institute). Statistical significance was set at a 2-sided *P* < .05. Data were analyzed from September 2022 through April 2023.

## Results

### Baseline Characteristics

Among 127 710 eligible patients included in the study (80 386 males [62.9%]; mean (SD) age, 62.6 [10.9] years), 37 308 patients were treated with VEGFR-TKIs and 90 402 patients were treated with capecitabine (eFigure 2 in Supplement 1). There were 4629 patients (12.4%) in the VEGFR-TKI group and 10426 patients (11.5%) in the capecitabine group enrolled in 2020 as the index year. After 1:1 PS matching, groups were evenly matched, with 27 535 patients in the VEGFR-TKI group (mean [SD] age, 62.9 [10.7] years; 20 170 males [73.3%]) and 27 535 patients in the capecitabine group (mean [SD] age, 63.1 [10.7] years; 19 818 males [72.0%]) (Table 1). We observed no significant difference between groups for any variables (standardized difference <10% [eg, 2.9% for sex]), and the distribution of PSs was confirmed visually (eFigure 3 in Supplement 1).

### Oral Intake of VEGFR-TKIs and Risk of AAD

We identified 478 patients newly diagnosed with AAD from 103 384.6 person-years of observations. AAD incidence rates were 5.7 and 4.3 per 1000 person-years among individuals prescribed VEGFR-TKIs and those prescribed capecitabine, respectively; the crude HR was 1.30 (95% CI, 1.07-1.59). Incidence rates of aortic aneurysms and dissections and AAD with rupture among patients receiving VEGFR-TKIs were 2.0 and 0.4 per 1000 person-years, respectively. Among patients treated with VEGFR-TKIs, the number of individuals experiencing AAD was the highest during the first 3 months (58 incidents), as recorded by the end of the follow-up; there were 40, 19, and 21 incidents during 3- to 6-month, 6- to 9-month, and 9- to 12-month periods, respectively.

After 1:1 PS matching, 109 and 95 patients were newly diagnosed with AAD in VEGFR-TKI and capecitabine groups, respectively (Table 2). The incidence of AAD within 1 year of treatment initiation was 6.0 per 1000 person-years. Incidence was highest in the first 3 months (45 incidents vs 31, 17, and 16 incidents during 3- to 6-month, 6- to 9-month, and 9- to 12-month periods, respectively). The median (IQR) time to the onset of AAD was 114 (67-257) and 120 (48-202) days for VEGFR-TKI and capecitabine groups, respectively. Patients receiving VEGFR-TKIs had a significantly higher risk of AAD incidence than those prescribed capecitabine (HR, 1.48; 95% CI, 1.08-2.02). There were significant differences between cumulative incidence curves over time (*P* = .01) (Figure, A). However, no significant differences were noted for incidence rates of aortic aneurysm and dissection (HR, 1.32; 95% CI, 0.81-2.16) or AAD with rupture (HR, 0.60; 95% CI, 0.22-1.65).

**Table 2.** Cox Regression Analysis After Propensity Score Matching

Outcome	Patients treated with VEGFR-TKI (n = 27 535)			Patients treated with capecitabine (n = 27 535)			HR (95% CI)
	Events, No.	Person-years	Incidence rate, No./1000 person-years	Events, No.	Person-years	Incidence rate, No./1000 person-years	
Primary outcome: AAD	109	18 122.9	6.0	95	23 319.5	4.1	1.48 (1.08-2.02)
Secondary outcomes							
Aortic aneurysm and dissection	41	18 147	2.3	40	23 343.4	1.7	1.32 (0.81-2.16)
AAD with rupture	7	18 162.1	0.4	10	23 360.7	0.4	0.60 (0.22-1.65)

Abbreviations: AAD, aneurysm and artery dissection; HR, hazard ratio; VEGFR-TKI, vascular endothelial growth factor receptor tyrosine kinase inhibitor.

**Figure.**
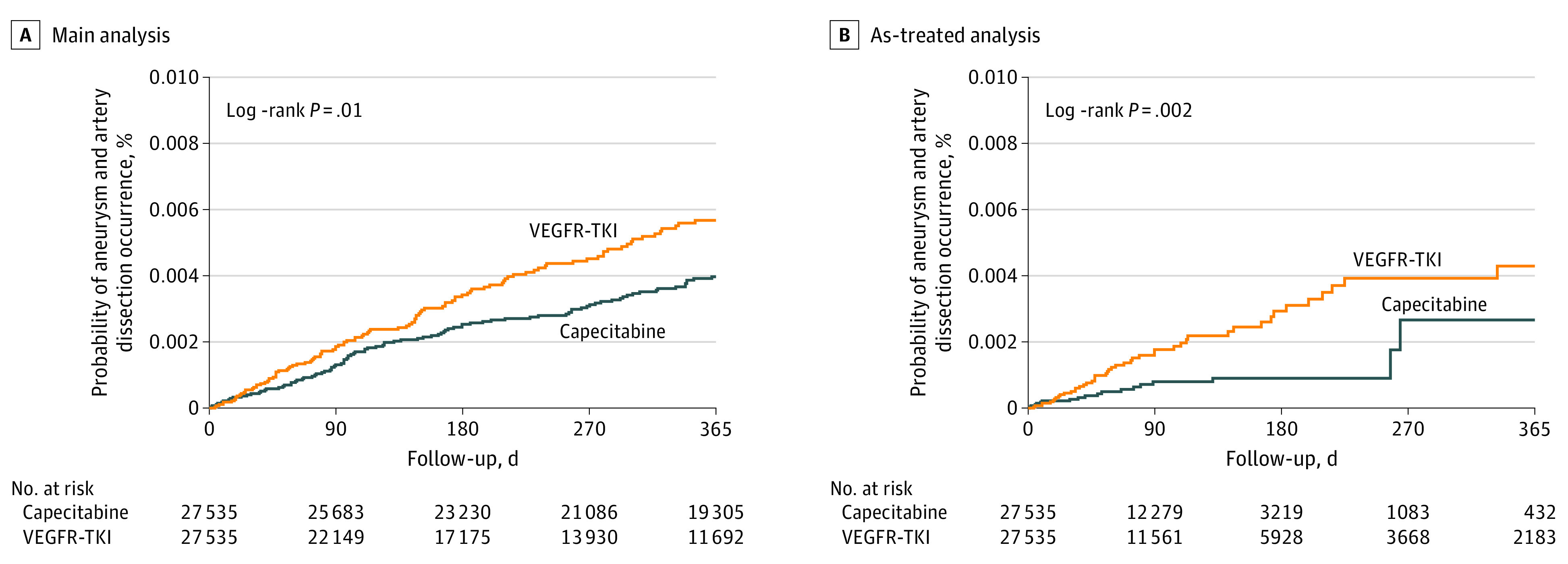
Kaplan-Meier Plots of Probability of Aneurysm and Artery Dissection Results of the survival analysis and log-rank test between patients receiving vascular endothelial growth factor receptor tyrosine kinase inhibitors (VEGFR-TKIs) and those receiving capecitabine from the main analysis after propensity score matching (A) and the as-treated analysis (B).

The incidence and risk of AAD were significantly higher among patients treated with VEGFR-TKIs than those treated with capecitabine in females (HR, 2.08; 95% CI, 1.26-3.42), older adults (aged ≥65 years; HR, 1.42; 95% CI, 1.01-1.99), and patients with dyslipidemia (HR, 1.58; 95% CI, 1.11-2.24) (Table 3), as well as among cancer subtypes (eTables 3 and 4 in Supplement 1). However, there were no significant differences between sexes, age groups, or dyslipidemia groups. We also analyzed baseline characteristics of patients categorized by sex (eTable 5 in Supplement 1). Females receiving VEGFR-TKIs were older and had a higher prevalence of underlying conditions, such as hypertension, than females receiving capecitabine.

**Table 3.** Subgroup Analysis

Outcome	Patients treated with VEGFR-TKI (n = 27 535)				Patients treated with capecitabine (n = 27 535)				HR (95% CI)	*P* for interaction
	Patients, No.	Events, No.	Person-years	Incidence rate, No./1000 person-years	Patients, No.	Events, No.	Person-years	Incidence rate, No./1000 person-years		
**Primary outcome: AAD**										
Sex										
Male	20 170	68	13 109.4	5.2	19 818	70	16 829.4	4.2	1.20 (0.86-1.68)	.07
Female	7365	41	5013.4	8.2	7717	25	6490.1	3.9	2.08 (1.26-3.42)	
Age, y										
<65	15 544	36	10 114.8	3.6	15 202	32	13 063.9	2.4	1.46 (0.90-2.35)	.92
≥65	11 991	73	8008	9.1	12 333	63	10 255.5	6.1	1.42 (1.01-1.99)	
History of hypertension										
No	13 683	35	8885.4	3.9	13 513	28	11 491.3	2.4	1.56 (0.95-2.56)	.67
Yes	13 852	74	9237.5	8	14 022	67	11 828.1	5.7	1.37 (0.99-1.91)	
History of type 2 diabetes										
No	15 905	54	10 520.9	5.1	15 979	50	13 645.2	3.7	1.39 (0.94-2.04)	.71
Yes	11 630	55	7601.9	7.2	11 556	45	9674.2	4.7	1.47 (0.99-2.19)	
History of dyslipidemia										
No	12 600	37	8273.5	4.5	12 726	39	10 732.5	3.6	1.21 (0.77-1.90)	.32
Yes	14 935	72	9849.4	7.3	14 809	56	12 586.9	4.4	1.58 (1.11-2.24)	
**Secondary outcomes**										
Aortic aneurysm and dissection										
Sex										
Male	20 170	31	13 121.9	2.4	19 818	33	16 844.3	2	1.16 (0.71-1.90)	.43
Female	7365	10	5025.1	2	7717	7	6499.1	1.1	1.81 (0.69-4.78)	
Age, y										
<65 y	15 544	9	10 123.3	0.9	15 202	9	13 071.9	0.7	1.27 (0.50-3.22)	.96
≥65 y	11 991	32	8023.7	4	12 333	31	10 271.5	3	1.28 (0.78-2.10)	
History of hypertension										
No	13 683	15	8893.5	1.7	13 513	10	11 498.9	0.9	1.89 (0.85-4.22)	.25
Yes	13 852	26	9253.5	2.8	14 022	30	11 844.4	2.5	1.07 (0.63-1.82)	
History of type 2 diabetes										
No	15 905	22	10 533.9	2.1	15 979	21	13 656.7	1.5	1.33 (0.73-2.42)	.88
Yes	11 630	19	7613.1	2.5	11 556	19	9686.7	2	1.22 (0.64-2.31)	
History of dyslipidemia										
No	12 600	18	8281.1	2.2	12 726	13	10 743.3	1.2	1.80 (0.88-3.67)	.27
Yes	14 935	23	9865.8	2.3	14 809	27	12 600.1	2.1	1.03 (0.59-1.80)	
AAD with rupture										
Sex										
Male	20 170	4	13 133.5	0.3	19 818	9	16 857.9	0.5	0.52 (0.16-1.70)	.11
Female	7365	3	5028.6	0.6	7717	1	6502.9	0.2	3.79 (0.39-36.64)	
Age, y										
<65	15 544	3	10 127.1	0.3	15 202	6	13 074.6	0.5	0.64 (0.16-2.56)	.48
≥65	11 991	4	8035	0.5	12 333	4	10 286.1	0.4	1.14 (0.29-4.57)	
History of hypertension										
No	13 683	1	8898.3	0.1	13 513	6	11 501.5	0.5	0.19 (0.02-1.61)	.05
Yes	13 852	6	9263.8	0.6	14 022	4	11 859.2	0.3	1.84 (0.52-6.55)	
History of type 2 diabetes										
No	15 905	3	10 542.1	0.3	15 979	6	13 664.9	0.4	0.59 (0.15-2.37)	.50
Yes	11 630	4	7620	0.5	11 556	4	9695.8	0.4	1.22 (0.30-4.89)	
History of dyslipidemia										
No	12 600	3	8285.5	0.4	12 726	5	10 747.8	0.5	0.71 (0.17-2.99)	.79
Yes	14 935	4	9876.6	0.4	14 809	5	12 612.9	0.4	0.97 (0.26-3.63)	

Abbreviations: AAD, aneurysm and artery dissection; HR, hazard ratio; VEGFR-TKI, vascular endothelial growth factor receptor tyrosine kinase inhibitor.

### Sensitivity Analyses

VEGFR-TKI use was associated with a higher risk of AAD compared with capecitabine use when changing the follow-up from 1 year to 9 months (HR, 1.53; 95% CI, 1.09-2.13), using an as-treated analysis (HR, 2.17; 95% CI, 1.09-4.29), and restricting the analysis to patients prescribed VEGFR-TKIs or capecitabine 2 or more times (HR, 1.63; 95% CI, 1.11-2.38) (Table 4). Time-dependent Cox regression models showed that the use of VEGFR-TKIs was a risk factor associated with AAD, even in patients with adjusted hypertension history; new-onset hypertension had no significant outcome (eTable 6 in Supplement 1). The *E* value was 2.32, indicating that an unmeasured confounder must have a risk ratio of 2.32 or greater to explain the observed association.

**Table 4.** Sensitivity Analysis

Outcome	Patients treated with VEGFR-TKI (n = 27 535)			Patients treated with capecitabine (n = 27 535)			HR (95% CI)
	Events, No.	Person-years	Incidence rate, No./1000 person-years	Events, No.	Person-years	Incidence rate, No./1000 person-years	
**Primary outcome: AAD**							
Observation period, mo							
3	45	6241.60	7.2	34	6606.80	5.1	1.39 (0.87-2.20)
6	76	11 025.20	6.9	63	12 627.30	5.0	1.42 (0.99-.04)
9	93	14 816.20	6.3	76	18 080.10	4.2	1.53 (1.09-2.13)
As-treated analysis	47	8559.60	5.5	18	7434.90	2.4	2.17 (1.09-4.29)
Prescription ≥2 times	92	16 476.20	5.6	71	20 907.60	3.4	1.63 (1.11-2.38)
**Secondary outcomes**							
Aortic aneurysm and dissection							
Observation period, mo							
3	18	6244.60	2.9	14	6609.00	2.1	1.42 (0.68-2.97)
6	30	11 034.00	2.7	25	12 635.50	2.0	1.33 (0.76-2.35)
9	36	14 831.80	2.4	32	18 095.10	1.8	1.36 (0.81-2.28)
As-treated analysis	22	8560.50	2.6	8	7435.10	1.1	2.75 (0.88-8.64)
Prescription ≥2 times	34	16 496.70	2.1	31	20 922.10	1.5	1.47 (0.79-2.72)
AAD with rupture							
Observation period, mo							
3	4	6245.90	0.6	4	6610.20	0.6	1.00 (0.25-4.00)
6	5	11 038.60	0.5	8	12 639.90	0.6	0.50 (0.15-1.66)
9	6	14 841.40	0.4	9	18 104.90	0.5	0.56 (0.19-1.66)
As-treated analysis	3	8561.40	0.4	1	7435.30	0.1	NA^a^
Prescription ≥2 times	7	6245.90	0.4	7	6609.00	0.3	0.86 (0.29-2.55)

Abbreviations: AAD, aneurysm and artery dissection; HR, hazard ratio; NA, not applicable; VEGFR-TKI, vascular endothelial growth factor receptor tyrosine kinase inhibitor.

^a^ Incidents and incidence rates were too small to obtain statistical values.

## Discussion

To the best of our knowledge, this cohort study is the first study to evaluate the incidence of AAD in patients treated with VEGFR pathway inhibitors based on clinical data from a nationwide health insurance service. The risk of AAD after the initiation of VEGFR-TKI treatment among patients with cancer increased 1.48-fold within 1 year compared with that among patients treated with capecitabine. The incidence of AAD within 1 year of initiating VEGFR-TKI treatment was 6.0 per 1000 person-years, with the highest incidence observed during the first 3 months.

Our results are consistent with those of previous studies that used spontaneous adverse event reporting databases to examine the association between VEGF pathway inhibitors and AAD. Oshima et al^14^ analyzed 11 years of data from the Japanese Adverse Drug Event Report database, finding that VEGF pathway inhibitors compared with non-VEGFR pathway inhibitors were associated with increased odds of aortic dissection (odds ratio, 22.3). Previous studies based on data from the US Food and Drug Administration Adverse Event Reporting System (FAERS) and World Health Organization-Uppsala Monitoring Centre VigiBase reported that VEGF pathway inhibitors were associated with a higher number of AAD incidents than other drugs^15,16^; however, these studies had limitations. Our study provides novel and robust evidence and clinical information, such as incidence rates, for the association between VEGFR-TKIs and AAD occurrence in cancer treatment and may be used as a reference for the safe use of VEGF pathway inhibitors.

The median time to the onset of AAD after VEGFR-TKI prescription was 114 days, consistent with previous findings ranging from 94 to 105 days.^14,15,16^ Although the absolute number of incidents was the highest during the 3 months after drug initiation, the slope of the survival curve remained the same for up to 1 year after drug initiation. This suggests that AAD may occur continuously regardless of the time of drug administration. Therefore, clinicians should consider the possibility of AAD incidence after the occurrence of symptoms, such as headache or abdominal pain, at any time after VEGFR-TKI administration.

The male-to-female ratio for the incidence of AAD ranged from 4:1 to 5:1 in previous studies,^25,39^ with females having a lower incidence owing to the inhibition of aneurysm development by estrogen. A previous study based on the FAERS database^15^ reported a higher incidence of VEGF pathway inhibitor–associated AAD in males; however, owing to limitations of the spontaneous reporting database, it was not possible to directly compare incidence rates by sex. Interestingly, the incidence rate of AAD in our study was higher in females than males despite the higher absolute number of incidents in males, and according to subgroup analysis, the risk of AAD was significantly higher in females treated with VEGFR-TKIs than in those treated with capecitabine. Consistent with our results, a 2023 analysis^40^ found that the risk for VEGFR-TKI–associated adverse events was significantly higher in females than males. However, a proportion of these patients were older than 70 years or had a history of hypertension, which are known risk factors associated with AAD. This complicates the definitive inference that AAD risk was higher in females than males receiving treatment with VEGFR-TKIs.^35,36,37^ Nevertheless, our findings have clinical implications and emphasize the need for careful monitoring for the possibility of AAD occurrence in females treated with VEGFR-TKIs.

We selected capecitabine as an active comparator to address potential confounding effects associated with drug indications and administration routes. Capecitabine, an oral anticancer drug independent of the VEGF pathway, is not associated with AAD incidence.^23^ Given the diverse risks of fluoroquinolone-induced AAD based on administration routes,^41^ we opted for capecitabine due to its comparable route of administration. Furthermore, capecitabine was used as a comparator in previous VEGFR-TKI trials.^42,43^ To the best of our knowledge, there are no reports finding an association between cancer subtype and AAD occurrence independent of therapy type. Despite distinct indications for cancer subtypes in the use of VEGFR-TKIs and capecitabine, we stratified subtypes. The incidence and risk of AAD with VEGFR-TKIs remained higher in gastrointestinal or stomach cancers.

Biological mechanisms underlying the increased risk of AAD after VEGFR-TKI administration have not yet been elucidated. The use of VEGFR-TKIs is hypothesized to increase hypertension incidence, thereby increasing the incidence of aortic dissection.^16^ However, in our study, new-onset hypertension had no association with the occurrence of AAD, including aortic dissection. One possible explanation is that VEGFR-TKIs disrupt the vascular wall integrity and cardiovascular homeostasis, causing AAD.^44^ Interestingly, this has been proposed as a biologically plausible mechanism for the association between the use of fluoroquinolones and granulocyte colony–stimulating factors and AAD occurrence.^17^ Contrary to previous clinical findings,^16^ a study in mice with induced abdominal aortic aneurysms proposed VEGFR-TKIs as novel aneurysm inhibitors; inhibiting VEGF activity using VEGF pathway inhibitors, such as sunitinib, prevented the development of abdominal aortic aneurysms and considerably reduced the diameter of existing aneurysms.^45^ Clinical evidence based on spontaneous adverse event reporting databases confirmed the increased risk of AAD occurrence after VEGFR-TKI treatment^14,15,16,17,18^; however, further studies are required to elucidate the mechanism underlying the association of VEGFR-TKIs with AAD.

### Limitations

This study has some limitations. First, we defined drug exposure based on patient prescription records; therefore, we could not verify actual drug intake. To address this, we performed a sensitivity analysis among patients who received prescriptions of study drugs at least twice and were most likely to have adhered to treatment, and this analysis supported the reliability of our results. Second, data were obtained from information accumulated for reimbursement purposes, which imposed certain inherent limitations. In particular, we had no access to detailed clinical information, such as smoking status, family history, genetic factors, cancer characteristics or stage of each patient, and the duration of cancer history, in matching variables. In addition, a time lag may have occurred between drug use and data construction; therefore, the possibility that we did not have access to all actual medical records cannot be excluded. However, participants who were admitted after 2020 accounted for approximately 12% of both groups; hence, the possibility of misclassification of outcome variables is expected to be nondifferential in both groups. Third, aneurysms included in the primary outcome may have been asymptomatic and were identified incidentally using computed tomography or ultrasonography; therefore, the occurrence of outcome variables may have been underestimated. Fourth, unmeasured confounders inherent in historical cohort studies cannot be excluded. The *E* value was 2.32, indicating that an unmeasured confounder with an HR of 2.32 or greater would have affected the exposure–outcome association and suggesting the robustness of our results. Fifth, only patients aged 40 years or older were included in this study; hence, further studies on younger patients remain warranted. Sixth, we investigated only the risks associated with drug use. However, given that patients with cancer often have limited treatment options, it is important to comprehensively consider the benefits and risks associated with drug use.

## Conclusions

In this cohort study of patients with cancer aged 40 years or older, the use of VEGFR-TKIs was associated with an increased risk of AAD occurrence. Our results may serve as a reference for regulatory decision-making, such as updating previous safety profiles. These findings may also contribute to the identification of the potential risk of aneurysm development associated with VEGFR-TKI use and early detection of its development through screening before the occurrence of complications, such as rupture, thus enhancing treatment efficacy and reducing socioeconomic losses.

## Notes

Supplement 1. **eTable 1.** Relevant ICD-10 Codes **eTable 2.** Covariate Definitions Based on ICD-10 or Anatomical Therapeutic Chemical Codes for Propensity Score Matching **eTable 3.** Number of Patients by Cancer Subtype After Matching **eTable 4.** Subgroup Analysis by Cancer Subtype **eTable 5.** Baseline Characteristics of Patients by Sex **eTable 6.** Adjusted Hazard Ratio of Time-Dependent Cox Regression Model for Patients With Hypertension **eFigure 1.** Study Design **eFigure 2.** Patient Inclusion Flowchart **eFigure 3.** Distribution of Log Odds Propensity Scores by Treatment Group

Supplement 2.
Data Sharing Statement
