# Supplementary material for: Aneurysm and Artery Dissection After Oral VEGFR-TKI Use in Adults With Cancer
Source: JAMA Netw Open. 2023 Nov 29;6(11):e2345977. doi: 10.1001/jamanetworkopen.2023.45977 (PMC10687660; doi:10.1001/jamanetworkopen.2023.45977)
Supplement: Supplement 1. — eTable 1. Relevant ICD-10 Codes eTable 2. Covariate Definitions Based on ICD-10 or Anatomical Therapeutic Chemical Codes for Propensity Score Matching eTable 3. Number of Patients by Cancer Subtype After Matching eTable 4. Subgroup Analysis by Cancer Subtype eTable 5. Baseline Characteristics of Patients by Sex eTable 6. Adjusted Hazard Ratio of Time-Dependent Cox Regression Model for Patients With Hypertension eFigure 1. Study Design eFigure 2. Patient Inclusion Flowchart eFigure 3. Distribution of Log Odds Propensity Scores by Treatment Group [file jamanetwopen-e2345977-s001.pdf]

Supplemental Online Content

Kang S, Yeon B, Kim M-S, Yoo M, Kim B, Yu YM. Aneurysm and artery dissection after oral VEGFR-TKI use in adults with cancer. *JAMA Netw Open*. 2023;6(11):e2345977. doi:10.1001/jamanetworkopen.2023.45977

eTable 1. Relevant ICD-10 Codes

eTable 2. Covariate Definitions Based on ICD-10 or Anatomical Therapeutic Chemical Codes for Propensity Score Matching

eTable 3. Number of Patients by Cancer Subtype After Matching

eTable 4. Subgroup Analysis by Cancer Subtype

eTable 5. Baseline Characteristics of Patients by Sex

eTable 6. Adjusted Hazard Ratio of Time-Dependent Cox Regression Model for Patients With Hypertension

eFigure 1. Study Design

eFigure 2. Patient Inclusion Flowchart

eFigure 3. Distribution of Log Odds Propensity Scores by Treatment Group

This supplemental material has been provided by the authors to give readers additional information about their work.

© 2023Kang S et al. *JAMA Network Open.*

eTable 1. Relevant ICD-10 codes

ICD-10 code	Names
Exclusion criteria	
Q25.1	Coarctation of aorta
Q87.4	Marfan's syndrome
Q22	Congenital malformations of pulmonary and tricuspid valves
Q23	Congenital malformations of aortic and mitral valves
Q79.6	Ehlers-Danlos syndrome
Q96	Turner's syndrome
Outcomes	
Primary outcomes: Aneurysm and artery dissection with or without rupture	
I71	Aortic aneurysm and dissection
I72	Other aneurysm and dissection
I77.2	Rupture of artery
I25.3	Aneurysm of heart
I25.4	Coronary artery aneurysm and dissection
I67.0	Dissection of cerebral arteries, nonruptured
I67.1	Cerebral aneurysm, nonruptured
Secondary outcomes: Aortic aneurysm and dissection	
I71	Aortic aneurysm and dissection
Secondary outcomes: Aneurysm and artery dissection with rupture	
I71.1	Thoracic aortic aneurysm, ruptured
I71.3	Abdominal aortic aneurysm, ruptured
I71.5	Thoracoabdominal aortic aneurysm, ruptured
I71.8	Aortic aneurysm of unspecified site, ruptured
I77.2	Rupture of artery

ICD, International Classification of Diseases

© 2023Kang S et al. *JAMA Network Open.*

eTable 2. Covariate definitions based on ICD-10 (for medical history) or ATC codes (for medication history) for propensity score matching

**Medical history**	**ICD-10 code**
Acute coronary syndrome	I200, I21-22
Other ischemic heart disease	I11 (not I110), I20 (not I200), I24, I25
Heart failure/cardiomyopathy	I110, I130, I132, I42, I43, I50, J81
Valve disorders	I34-37
Cerebrovascular diseases	G45, G46, I60-69
Arterial diseases	I65, I70, I72-I77, K550, K551
Arrhythmia	I44-49
Lung diseases	I27, J84, R092, E662, J40-47, J60-70, J92, J96, J982, J983; procedure code GGB
Liver diseases	B18, I850, I859, I982, K70-K77
Renal diseases	I12, I13, N00-08, N17, N18, N19, N25, Z49
Rheumatic disease	M05-09, M30-34, M351, M353, M45
Psychiatric disorders	F04-09, F20-99
Hypertension	I10, I11-I13, I15
Diabetes mellitus	E10-E14
Dyslipidemia	E78
Trauma	S00-S99, T00-T35
Drug abuse	F11-F16, F18, F19, Z715, Z722
Obesity	E66
**Medication history**	**ATC code**
ACE inhibitors or ARBs	C09A-D
Calcium channel blockers	C08C, C08D
Loop diuretics	C03C, C03EB
Other diuretics	C03A, C03B, C03D, C03EA
Beta-blocker	C07
Digoxin	C01AA05
Nitrate	C01DA
Platelet inhibitor	B01AC
Anticoagulant	B01AA
Lipid-lowering drugs	C10
Antidiabetic drug (not insulin)	A10B
Insulin	A10A
Antidepressant	N06A
Antipsychotic	N05A
Anxiolytic, hypnotic, or sedative	N05B, N05C
Corticosteroid for systemic use	H02
Nonsteroidal anti-inflammatory drug	M01A
Opiate	N02A
Systemic hormone replacement therapy	G03CA03, G03CA04, G03CA53, G03CA57, G03FA01, G03FA11, G03FA12, G03FA15, G03FA17, G03FB01, G03FB05, G03FB06, G03FB09, G03FB11, G03HB01, G03XC01, G03DC05, G03CX01
Fluoroquinolone	J01MA
Granulocyte colony-stimulating factor	L03AA
Fibrinolytic drug	B01AD
**History of anticancer therapy**	**ATC or insurance code**
Cytotoxic therapy	L01AA01, L01AA02, L01AA03, L01AA06, L01AA09, L01AB01, L01AC01, L01AD06, L01AX03, L01AX04, L01BA01, L01BA04, L01BB02, L01BB03 , L01BB04, L01BB05, L01BB06, L01BC, L01BC, L01BC01, L01BC02, L01BC04, L01BC05, L01BC07, L01BC08, L01BC53, L01BC53, L01CA01, L01CA02, L01CA04, L01CB01, L01CD01, L01CD02, L01CD04, L01CE01, L01CE02, L01CE04, L01DA01, L01DB01, L01DB02, L01DB03, L01DB06, L01DB07, L01DB08, L01DC01, L01DC03, L01XA, L01XA01, L01XA02, L01XA03, L01XX01, L01XX03, L01XX41

Targeted therapy	L01EF03, L01EB03, L01ED03, L01XG01, L01FX05, L01ED04, L01XG02, L01ED02, L01XC06, L01ED01, L01EC02, L01EB07, L01XC24, L01EB02, L01EG02, L01EB01, L01EL01, L01EA01, L01FB01, L01EH01, L01EB09, L01EA03, L01XK02, L01XC15, L01XK01, L01FX10, L01EB06, L01EB04, L01EF01, L01XC13, L01EA, L01EF02, L01XC02, L01EG01, L01EE01, L01XC03, L01EC01
Hormone therapy	L02BX03, L02BG03, L02BB03, L02BX02, L02BB04, L02BG06, L02BA03, L02AE03, L02BG04, L02AE02, L02BA01, L02BA02, L02AE04
Immunotherapy	L01XC32, L01XC31, L01XC19, L01XC28, L01XC11, L01XC17, L01XC18
Others	L03AC01, L01XX27, L01XX11, L01XX05, L03AB04, L03AB05, L01XG03, L01XX02, L04AX04, L04AX06, L04AC11, L04AX02, L01XF01, L01XX, L01XX52
Radiation therapy	Insurance codes associated with the radiation therapy (HD010-23, HD031-33, HD040-41, HD051-61, HD071-89, HD091-93, HD110-115, HD121, HD150, HD160, HD170, HD211-212, HD410-416, HD418-420, HD441, HX401, HY402, HY404, HY405, HZ271)

ATC, anatomical therapeutic chemical; ICD, International Classification of Diseases

© 2023Kang S et al. *JAMA Network Open.*

eTable 3. Number of patients according to cancer subtype after matching

Cancer subtype	ICD-10 code	Patients treated with VEGFR-TKIs, n (%)	Patients treated with capecitabine, n (%)
Digestive organs	C15–26	18 709 (68.4)	26 416 (96.6)
Stomach	C16	464 (1.7)	7866 (28.8)
Small intestine	C17	379 (1.4)	177 (0.6)
Colon	C18	223 (0.8)	6669 (24.4)
Rectum	C19–20	105 (0.4)	7727 (28.2)
Liver and intrahepatic bile ducts	C22	17 035 (62.3)	868 (3.2)
Gallbladder and biliary tract	C23–24	45 (0.2)	1067 (3.9)
Pancreas	C25	302 (1.1)	1468 (5.4)
Others	C15, C21, C26	156 (0.6)	574 (2.1)
Respiratory and intrathoracic organs	C30–39	397 (1.5)	126 (0.5)
Bone and articular cartilage	C40–41	123 (0.4)	9 (0)
Skin	C43–44	73 (0.3)	15 (0.1)
Mesothelial and soft tissue	C45–49	1297 (4.7)	31 (0.1)
Breast	C50	71 (0.3)	2532 (9.3)
Genital organs	C51–63	627 (2.3)	181 (0.7)
Female	C51–58	386 (1.4)	40 (0.1)
Male	C60–63	241 (0.9)	141 (0.5)
Urinary tract	C64–68	6023 (22.0)	76 (0.3)
Kidney and renal pelvis	C64–65	5957 (21.8)	31 (0.1)
Others	C66–68	66 (0.2)	45 (0.2)
Eye, brain and other parts of central nervous system	C69–72	65 (0.2)	11 (0)
Thyroid and other endocrine glands	C73–75	1198 (4.4)	90 (0.3)
Thyroid	C73	1169 (4.3)	77 (0.3)
Others	C74–75	29 (0.1)	13 (0)
Other sites	C00–14, C76	74 (0.3)	38 (0.1)

© 2023Kang S et al. *JAMA Network Open.*

eTable 4. Subgroup analysis according to cancer subtype

Outcomes	Patients treated with VEGFR-TKIs (N = 27 535)				Patients treated with capecitabine (N = 27 535)				
	**Patients, *n***	**Events, *n***	**P-Y**	**Incidence rate (per 1000 P-Y)**	**Patients, *n***	**Events, *n***	**P-Y**	**Incidence rate (per 1000 P-Y)**	**HR (95 % CI) [a]**
**Aneurysm and artery dissection**									
Cancer subtype (ICD-10 code)									
GI tract (C16–20)	1320	4	889	4.4	31	0	22	0	**2.84 (.41–5.49)**
Stomach (C16)	464	6	342.8	17.5	7866	19	6563.2	2.9	**6.03 (2.41–15.12)**
Liver (C22)	17 035	52	9892.9	5.3	868	7	584.8	12	**0.43 (0.20–0.96)**

CI, confidence interval; pts, patients; P-Y, person-years; GI, gastrointestinal

[a]Bold indicates statistical significance.

© 2023Kang S et al. *JAMA Network Open.*

eTable 5. Baseline characteristics of patients according to sex (N = 55 070)

Variables	Men			Women		
	Patients treated with VEGFR-TKIs (N=20 170, 73.3 %)	Patients treated with capecitabine (N=19 818, 72.0 %)	*P*[a]	Patients treated with VEGFR-TKIs (N=7365, 26.7 %)	Patients treated with capecitabine (N=7717, 28.0 %)	*P*[a]
	n (%)	n (%)		n (%)	n (%)	
Age, mean (SD)	62.3 (10.5)	63.6 (10.2)	**<.01**	64.5 (11.1)	61.9 (11.8)	**<.01**
40–49 years	2398 (11.9)	1727 (8.7)	**<.01**	769 (10.4)	1330 (17.2)	**<.01**
50–59 years	6091 (30.2)	5497 (27.7)		1733 (23.5)	2241 (29.0)	
60–69 years	6238 (30.9)	6510 (32.8)		2193 (29.8)	1863 (24.1)	
≥70 years	5443 (27.0)	6084 (30.7)		2670 (36.3)	2283 (29.6)	
Medical history						
Acute coronary syndrome	829 (4.1)	826 (4.2)	.77	216 (2.9)	189 (2.4)	.07
Other ischemic heart disease	3029 (15.0)	3063 (15.5)	.22	1039 (14.1)	964 (12.5)	**<.01**
Heart failure/cardiomyopathy	1721 (8.5)	1754 (8.9)	.26	753 (10.2)	696 (9.0)	**.01**
Valve disorders	76 (0.4)	91 (0.5)	.20	52 (0.7)	40 (0.5)	.14
Cerebrovascular diseases	1937 (9.6)	1958 (9.9)	.35	688 (9.3)	666 (8.6)	.13
Arterial diseases	2570 (12.7)	2711 (13.7)	.01	1015 (13.8)	885 (11.5)	**<.01**
Arrhythmia	1286 (6.4)	1315 (6.6)	.29	450 (6.1)	395 (5.1)	**.01**
Lung diseases	7213 (35.8)	7407 (37.4)	**<.01**	2887 (39.2)	2981 (38.6)	.47
Liver diseases	14 893 (73.8)	13 991 (70.6)	**<.01**	4915 (66.7)	5180 (67.1)	.61
Renal diseases	1984 (9.8)	2024 (10.2)	.21	617 (8.4)	652 (8.4)	.88
Rheumatic disease	742 (3.7)	693 (3.5)	.33	441 (6.0)	481 (6.2)	.53
Psychiatric disorders	5493 (27.2)	5550 (28.0)	.09	2703 (36.7)	2740 (35.5)	.13
Hypertension	9938 (49.3)	10 389 (52.4)	**<.01**	3914 (53.1)	3633 (47.1)	**<.01**
Diabetes mellitus	8716 (43.2)	8759 (44.2)	**.05**	2914 (39.6)	2797 (36.2)	**<.01**
Dyslipidemia	10 891 (54.0)	10 737 (54.2)	.72	4044 (54.9)	4072 (52.8)	**.01**
Trauma	7111 (35.3)	7185 (36.3)	**.04**	2972 (40.4)	3023 (39.2)	.14
Drug abuse	14 (0.1)	12 (0.1)	.73	7 (0.1)	6 (0.1)	.72
Obesity	7 (0)	9 (0)	.60	6 (0.1)	4 (0.1)	.54
Medication history						
ACE inhibitors or ARBs	6293 (31.2)	6591 (33.3)	**<.01**	2326 (31.6)	2116 (27.4)	**<.01**
Calcium channel blockers	7847 (38.9)	8244 (41.6)	**<.01**	3026 (41.1)	2734 (35.4)	**<.01**
Loop diuretics	6001 (29.8)	6263 (31.6)	**<.01**	2571 (34.9)	2402 (31.1)	**<.01**
Other diuretics	4335 (21.5)	4499 (22.7)	**<.01**	1960 (26.6)	1851 (24.0)	**<.01**
Beta-blockers	4857 (24.1)	4840 (24.4)	.43	1662 (22.6)	1590 (20.6)	**<.01**
Digoxin	175 (0.9)	177 (0.9)	.79	79 (1.1)	76 (1.0)	.60
Nitrates	2272 (11.3)	2404 (12.1)	**.01**	554 (7.5)	480 (6.2)	**<.01**
Platelet inhibitors	4324 (21.4)	4429 (22.3)	**.03**	1356 (18.4)	1273 (16.5)	**<.01**
Anticoagulants	6402 (31.7)	6028 (30.4)	**<.01**	2301 (31.2)	2283 (29.6)	**.03**
Lipid-lowering drugs	4206 (20.9)	4201 (21.2)	.40	1705 (23.2)	1773 (23.0)	.80
Antidiabetic drugs (not insulin)	4852 (24.1)	4922 (24.8)	.07	1508 (20.5)	1461 (18.9)	**.02**
Insulin	3655 (18.1)	3659 (18.5)	.38	1113 (15.1)	1053 (13.6)	**.01**
Antidepressants	2297 (11.4)	2301 (11.6)	.49	1259 (17.1)	1283 (16.6)	.44

Antipsychotics	1049 (5.2)	1080 (5.4)	.27	391 (5.3)	415 (5.4)	.85
Anxiolytic, hypnotic, or sedatives	13 120 (65.0)	12 561 (63.4)	**<.01**	4729 (64.2)	4672 (60.5)	**<.01**
Corticosteroids for systemic use	11 253 (55.8)	10 741 (54.2)	**<.01**	4689 (63.7)	5528 (71.6)	**<.01**
NSAIDs	16 114 (79.9)	15 839 (79.9)	.94	6237 (84.7)	6612 (85.7)	.09
Opiates	16 981 (84.2)	16 543 (83.5)	.05	6312 (85.7)	6543 (84.8)	.11
Systemic HRT	10 (0)	7 (0)	.49	242 (3.3)	260 (3.4)	.78
Fluoroquinolones	5628 (27.9)	5557 (28.0)	.76	2492 (33.8)	2604 (33.7)	.91
G-CSF	556 (2.8)	506 (2.6)	.21	640 (8.7)	779 (10.1)	**<.01**
Fibrinolytic drugs	54 (0.3)	57 (0.3)	.71	36 (0.5)	34 (0.4)	.66
Anticancer therapy						
Cytotoxic agents	6577 (32.6)	5655 (28.5)	**<.01**	2716 (36.9)	3612 (46.8)	**<.01**
Targeted anticancer drugs	820 (4.1)	433 (2.2)	**<.01**	505 (6.9)	1029 (13.3)	**<.01**
Hormone treatment	111 (0.6)	31 (0.2)	**<.01**	82 (1.1)	235 (3.0)	**<.01**
Cancer immunotherapy	6 (0)	8 (0)	.57	5 (0.1)	1 (0)	.12
Others	38 (0.2)	45 (0.2)	.40	23 (0.3)	11 (0.1)	**.03**
Radiation therapy	3127 (15.5)	2740 (13.8)	**<.01**	1428 (19.4)	1911 (24.8)	**<.01**

ACE, angiotensin-converting enzyme; ARB, angiotensin II receptor blocker; G-CSF, granulocyte colony-stimulating factor; HRT, hormone replacement therapy; NSAIDs, nonsteroidal anti-inflammatory drugs

[a]Bold indicates statistical significance.

eTable 6. Adjusted hazard ratio of the time-dependent Cox regression model for patients with HTN

Variables	aHR (95 % CI)	*P*
VEGFR-TKI (ref. capecitabine)	1.47 (1.12–1.93)	.01
Previous HTN (ref. no HTN)[a]	1.90 (1.40–2.57)	<.01
New-onset HTN (ref. no HTN)[a]	1.57 (0.89–2.74)	.12

aHR, adjusted hazard ratio; CI, confidence interval; HTN, hypertension

[a]No HTN refers to patients who had not been diagnosed with hypertension between 1 year before the index date and the end date of follow-up. Previous HTN refers to patients who had been diagnosed with hypertension within 1 year before the index date. New-onset HTN refers to patients who were diagnosed with hypertension between the index date and the end date of follow-up.

© 2023Kang S et al. *JAMA Network Open.*

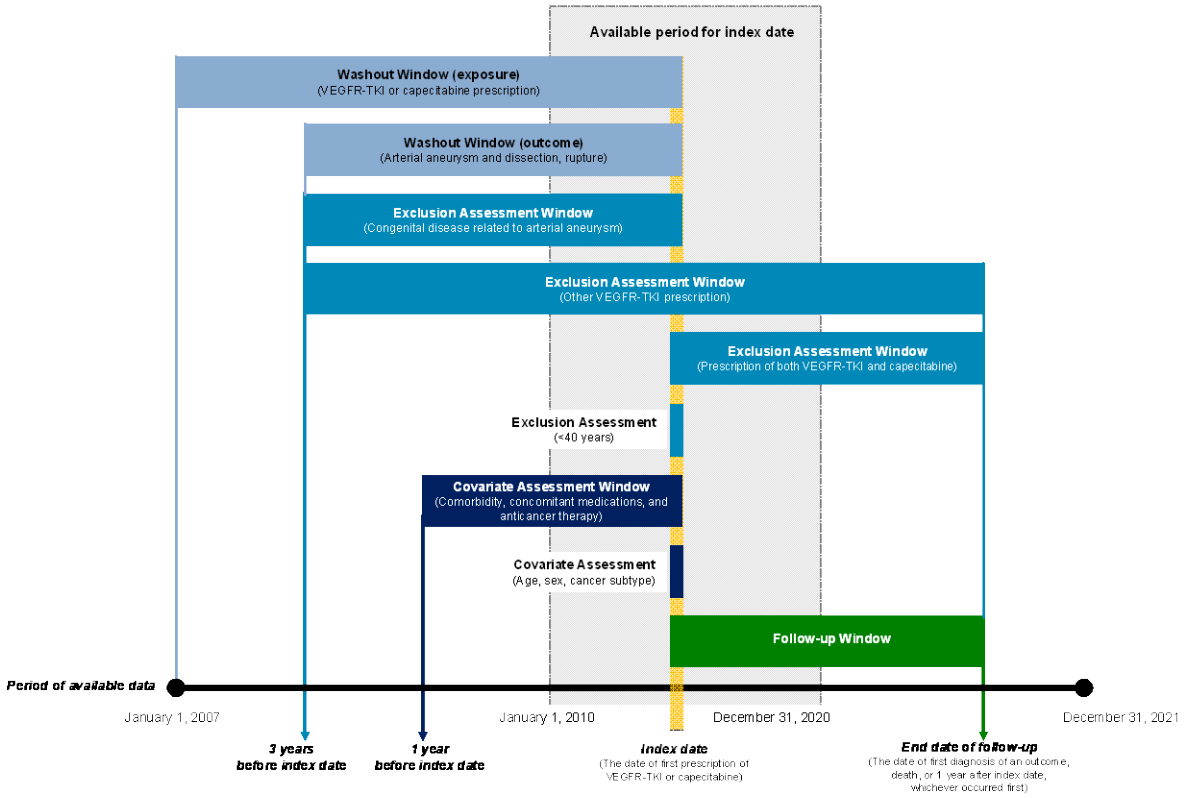

eFigure 1. Study design

Black solid line indicates time line of available data period.

© 2023Kang S et al. *JAMA Network Open.*

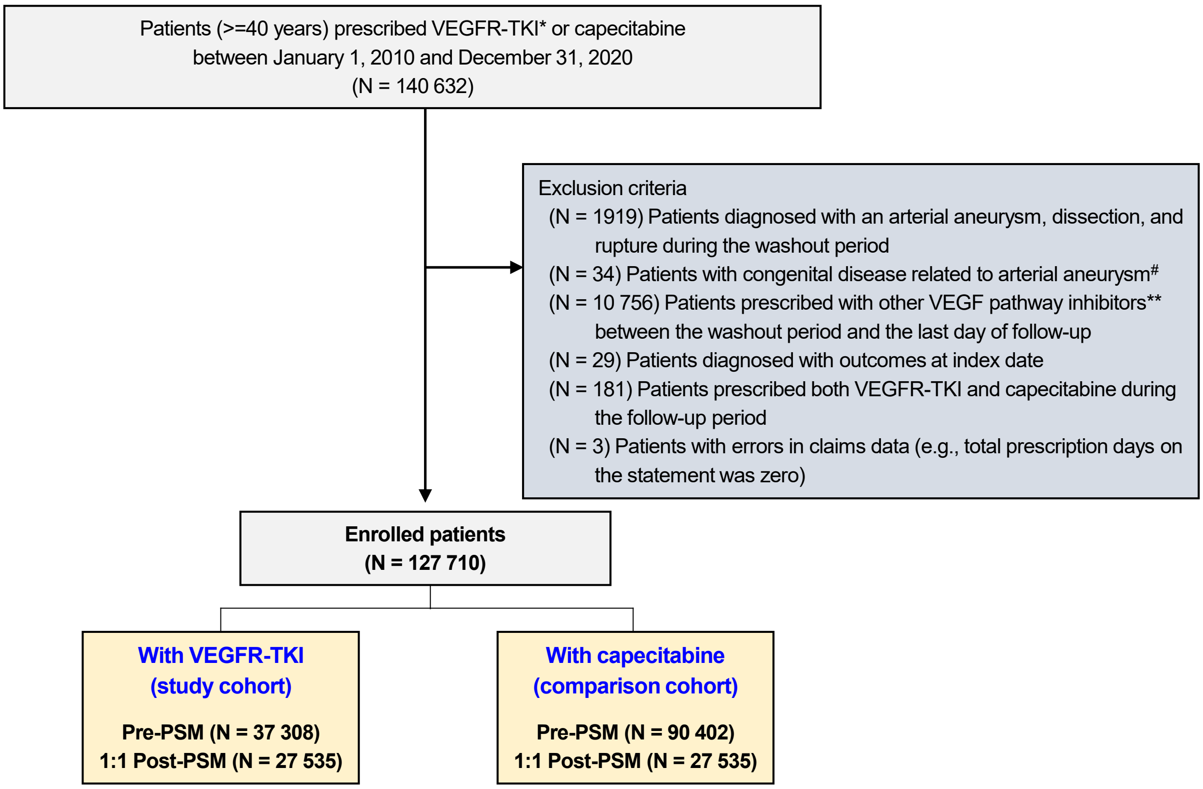

* Sorafenib, regorafenib, vandetanib, sunitinib, lenvatinib, axitinib, pazopanib

Coarctation of the aorta, Marfan syndrome, congenital malformations of pulmonary valve, tricuspid valve, aortic and mitral valves, Ehlers-Danlos syndrome, Turner's syndrome

** Bevacizumab, ziv-aflibercept, ramucirumab, cabozantinib, ponatinib, ranibizumab, nintedanib, brolucizumab

eFigure 2. Patient inclusion flowchart

© 2023Kang S et al. *JAMA Network Open.*

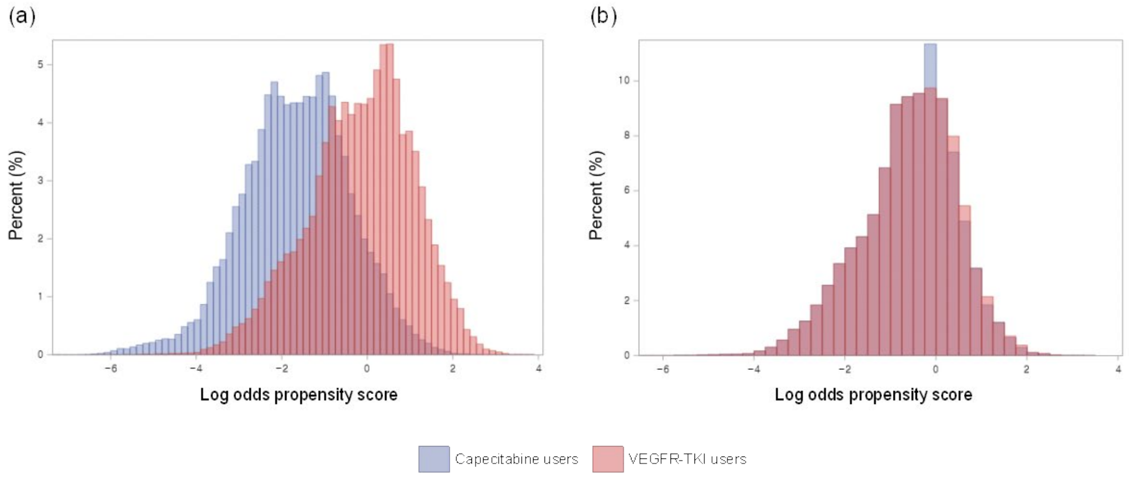

eFigure 3. Distribution of log odds propensity scores between patients treated with VEGFR-TKIs and those treated with capecitabine (a) before and (b) after matching

© 2023Kang S et al. *JAMA Network Open.*
