# Supplementary material for: Aneurysm and Artery Dissection After Oral VEGFR-TKI Use in Adults With Cancer
Source: JAMA Netw Open. 2023 Nov 29;6(11):e2345977. doi: 10.1001/jamanetworkopen.2023.45977 (PMC10687660; doi:10.1001/jamanetworkopen.2023.45977)
Supplement: Supplement 2. — Data Sharing Statement [file jamanetwopen-e2345977-s002.pdf]

Data Sharing Statement

Kang. Aneurysm and Artery Dissection After Oral VEGFR-TKI Use in Adults With Cancer. *JAMA Netw Open*. Published online November 29, 2023. doi:10.1001/jamanetworkopen.2023.45977

Data
Data available: No
